# Modelling the Response of FOXO Transcription Factors to Multiple Post-Translational Modifications Made by Ageing-Related Signalling Pathways

**DOI:** 10.1371/journal.pone.0011092

**Published:** 2010-06-14

**Authors:** Graham R. Smith, Daryl P. Shanley

**Affiliations:** Henry Wellcome Laboratory for Biogerontology, Institute for Ageing and Health, Newcastle University, Newcastle upon Tyne, United Kingdom; Virginia Commonwealth University, United States of America

## Abstract

FOXO transcription factors are an important, conserved family of regulators of cellular processes including metabolism, cell-cycle progression, apoptosis and stress resistance. They are required for the efficacy of several of the genetic interventions that modulate lifespan. FOXO activity is regulated by multiple post-translational modifications (PTMs) that affect its subcellular localization, half-life, DNA binding and transcriptional activity. Here, we show how a mathematical modelling approach can be used to simulate the effects, singly and in combination, of these PTMs. Our model is implemented using the Systems Biology Markup Language (SBML), generated by an ancillary program and simulated in a stochastic framework. The use of the ancillary program to generate the SBML is necessary because the possibility that many regulatory PTMs may be added, each independently of the others, means that a large number of chemically distinct forms of the FOXO molecule must be taken into account, and the program is used to generate them. Although the model does not yet include detailed representations of events upstream and downstream of FOXO, we show how it can qualitatively, and in some cases quantitatively, reproduce the known effects of certain treatments that induce various single and multiple PTMs, and allows for a complex spatiotemporal interplay of effects due to the activation of multiple PTM-inducing treatments. Thus, it provides an important framework to integrate current knowledge about the behaviour of FOXO. The approach should be generally applicable to other proteins experiencing multiple regulations.

## Introduction

The FOXO (Forkhead Box, type O) family of transcription factors (TFs) cause changes in gene expression to implement a cellular stress response programme, and an increase in their activity is a consequence of many of the genetic interventions that extend lifespan in model organisms. FOXO transcription factors are active when an organism is fasting, whereas feeding causes the activation of the Insulin- and Insulin-like Signalling (IIS) pathway, and in particular of Akt/PKB, which negatively regulates FOXO TFs by phosphorylations that cause translocation to the cytoplasm, thus suppressing their transcriptional activity. However, the location, synthesis/degradation and transcriptional behaviour can all be modified by signals from a variety of cellular signalling pathways, which are integrated by FOXO as a net response to the total set of modifications (phosphorylations, acetylations and ubiquitinations), that it undergoes. Integrating this knowledge within an extensible framework would provide a valuable means to organise the information and to explore the role of the modifications.

FOXO transcription factors are highly evolutionarily conserved in multicellular animals. There are four closely related FOXO paralogues in mammals, FOXO1 (formerly FKHR), FOXO3 (formerly FKHRL1; often known as FOXO3A), FOXO4 (formerly AFX) and FOXO6 (for the human genes, Ensembl accession codes ENSG00000150907, ENSG00000118689, ENSG00000184481 and ENSG00000204060, and for human proteins, UniProt accession codes Q12778, O43524, P98177 and A8MYZ6, respectively). All are ubiquitously expressed, but FOXO1 is particularly highly expressed in adipose tissue, FOXO3 in the brain, FOXO4 in the heart, and FOXO6 in the developing brain [1]. FOXO1 is particularly associated with the regulation of metabolism [2] and homozygous deletion is embryonic lethal, while FOXO3 homozygous knockout mice are viable but infertile, and FOXO4 deletions are also viable with no apparent phonotype. In *Caenorhabditis elegans* the FOXO orthologue is produced from the daf-16 gene (wormbase WBGene00000912). In *Drosophila melanogaster*, there is again a single dFOXO gene (flybase FBgn0038197). Proteins in the FOXO family are more distantly related to other FOX-domain containing TFs, such as the FOXA family, which themselves have a role in ageing [3]. The genes regulated by FOXO family members have been studied, both by high-throughput methods (mainly microarrays ([4]–[13] but also proteomics [14]) and studies concentrating on one or a few genes. In mammals, these include Bim [15]–[18]; MnSOD (SOD2) [19]; GADD45 [20]; p21cip1/waf1 (CDKN1A) [21]; p15ink4b (CDKN2B) [22]; CITED-2 (involving interactions with HIF) [23]; p27kip1 (CDKN1B) [16], [19], [24], [25]; autophagy [26]; IGFBP-1 [27], [28]; IRS2 [28], SIRT1 (also involving P53) [29]; and InsR [30]; in *C elegans*: MnSOD (SOD2, SOD3 in mammals), [19], [31]; daf-15 (raptor) [32]; gadd45, p27kip1, and FasL [33], [34]. FOXO TFs may also regulate their own expression [35], [36] as well as being under the control of E2F1 [37]. Their action as TFs may be modulated by association with other factors such as p53 (in the regulation of SIRT1 [29]), RunX3 (in the regulation of Bim) [18] and Smad 3/4 and FOXG (in the regulation of p21cip1/waf1) [21], [22]. Indeed, through these associations, FOXOs may influence transcription independently of DNA binding. The individually studied FOXO-regulated genes tend to fall into categories such as stress resistance (SOD2, catalase), negative regulation of the cell cycle (p27kip1, GADD45), and apoptosis (FasL, Bim). A comparative genomics study [38] of microarray data from flies, worms and mice has shown that, while there appears to be little conservation of particular orthologous genes, at least between mammals and invertebrates, there is consistent down-regulation of genes associated with protein biosynthesis and up-regulation of genes associated with sugar catabolism, energy generation, and several categories of cellular detoxification. Additionally, single-gene studies have shown up some further cross-species similarities e.g. InsR, p27kip1, and Bim.

**Figure 1:**
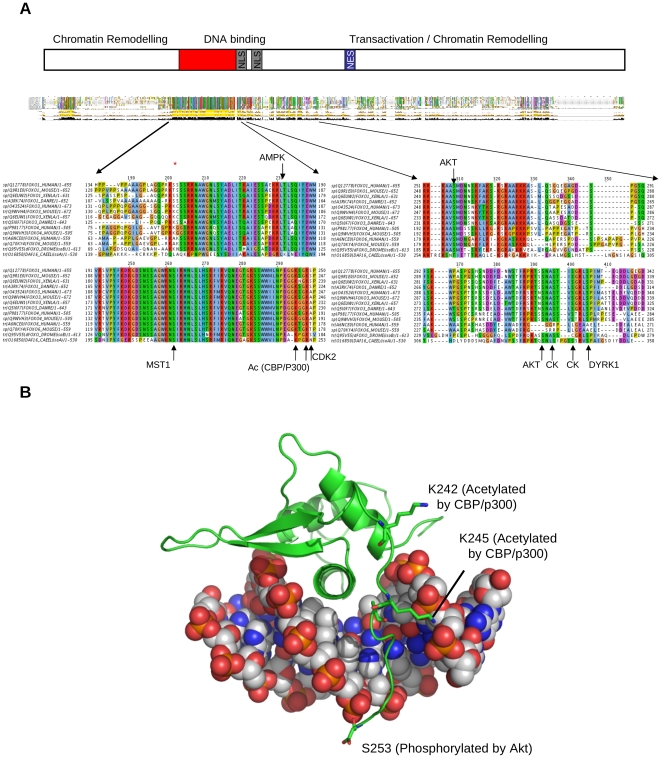
Sequence alignment and structure of FOXO. A: Multiple sequence alignment of FOXOs from human, mouse, zebrafish, Xenopus, Drosophila, C elegans, together with a schematic diagram based on figure 2 of Calnan and Brunet 2008 [54]. NES = Nuclear Export Sequence; NLS = Nuclear Localization Sequence.The Forkhead domain is expanded and conserved regulatory sites are indicated. The alignment was made with T-Coffee [136] and the graphic made with Jalview [137]. B: FOXO3 bound to DNA; 2UZK [39]. The graphic was made with PyMOL (DeLano Scientific, Palo Alto, CA, USA).

In Figure 1A, we show a multiple sequence alignment of FOXO family members from human (*Homo sapiens*), mouse (*Mus musculus*), *Xenopus leavis*, *Dario rerio*, Drosophila and *C elegans*. There are some regions of conservation throughout, especially in the DNA-binding domain (expanded region). Some of the modifiable sites in this domain are highlighted, along with an indication of the enzyme that modifies them. It is clear that there a great many regulators of FOXO (among them Akt, CDK2, CK1, JNK, IKK, AMPK, SIRT1 and CBP/P300), and a great many modifiable sites. The multiple sequence alignment is available as supplementary dataset S1. In several cases, the modifiable amino acid contacts DNA directly, making it obvious how a covalent change could alter DNA-binding behaviour; this is shown in Figure 1B, a representation of the structure of Human FOXO3 bound to DNA (PDB accession 2UZK [39]); more recently, the effect of acetylation on binding has been shown directly [40].

FOXO is implicated in many of the interventions that extend lifespan in worms, flies and mice, particularly genetic interventions in the IIS pathway and some protocols of dietary restriction (DR). Overexpression of FOXO itself extends lifespan moderately in flies [41], [42], as do the overexpression of the regulators JNK in flies [43] and worms [44], MST in worms [45], AMPK in worms [10], and sir2.1 in worms [46]. Lifespan is extended by many loss-of-function mutations of daf-2, (the C elegans insulin-like receptor) and other molecules in the IIS pathway in a FOXO/daf-16 dependent manner. Similarly, lifespan is extended by DR in ways that, depending on the protocol, may be dependent on FOXO (e.g sDR, [10]), partially dependent on it (IF, [47]) or independent of it [48]. See Greer et al. [49] for a recent review of methods of DR in C elegans. Certain FOXO hapolotypes [50]–[52] and SNPs in the FOXO3 gene have been found to be associated with human longevity [53], although interestingly the SNPs are all found in intronic regions, and do not seem to introduce or remove sequence features related to splicing, so it is not clear how they have their effect.

Many reviews of the behaviour and role of FOXO are available, related to its PTMs [54], its role in ageing and the maintenance of homeostasis [55], [56], metabolism [2], cancer and apoptosis [57], the immune system [58], cancer [59], ageing and cancer [1], its structure/function relationships [60], interaction partners [61], and subcellular localization and transcription [62]. Karpac and Jasper [63] review the interaction between IIS and stress signalling through JNK, while van der Horst et al [64] and Levine et al [65] review the interaction with stress signalling more generally, and compare and contrast the roles of FOXO and p53. What is clear is that the interpretation of the multiple complex regulations and interactions will be much assisted by a quantitative modelling approach.

To our knowledge FOXO has not been previously the subject of computational modeling, though other transcription factors have, for example the SMAD2/SMAD4 complex [66], MSN2/4 [67], [68], NF-κB [69], p53 [70], [71]. The forkhead homologue from fission and budding yeast, FKH2, has been treated as part of a wide survey of regulation of the cell cycle by coupled transcription and phophorylation [72], though its regulation was not modelled in detail. Several of the above references include the modelling of nuclear-cytoplasmic shuttling, which is a key regulatory mechanism of many TFs, including FOXO.

It is clear that FOXO has important roles in ageing, nutrient response and stress response pathways; and also that the large number of regulations that it undergoes and their different and often contradictory effects, make it difficult to understand the system. Hence, we believe that it is a good candidate for computational modelling, which, by integrating information from diverse sources and making possible a quantitative description, has the ability to resolve some of these difficulties [73]. We have produced a model in which the level of various modifiers of FOXO (activities of modifying enzymes) are set as inputs, and in which the behaviour of the FOXO in terms of localization, protein level and the transcriptional response are the outputs. Because of the complexity of the interaction of FOXO PTMs, this already produces interesting behaviour, and provides the core of future models in which the concentrations and activities of the modifying enzymes will themselves be dynamic variables, set by extracellular signals or by feedback regulation due, directly or indirectly, to genes regulated by FOXO, and the transcriptional output will include the response of multiple genes.

## Methods

There are two problems to be faced in modelling the regulation of FOXO. Firstly, it is necessary to choose a model structure that simplifies the large (and still not fully elucidated) set of PTMs, while still retaining enough information to describe all the relevant regulations. Secondly, appropriate parameters must be set for the reactions, even though detailed kinetic measurements are often not available.

**Figure 2:**
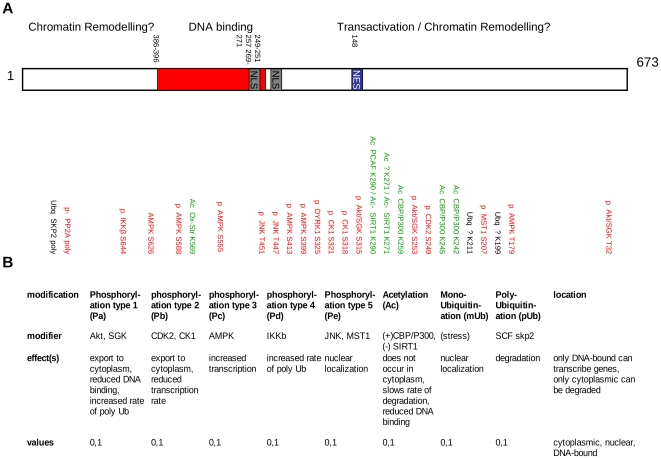
Summary of FOXO PTMs. A: Sites and types of PTMs; and modifying enzymes; based on figure 2 of Calnan and Brunet 2008 [54]. B: Simplified set of collective PTMs and their effects.

The first problem is a consequence mainly of the large number of independent PTMs, which lead to a need to include many species to describe all the combinations. This can be appreciated by considering Figure 2A, which shows a fairly complete (but not exhaustive) list of the known modifiable residues of mammalian FOXOs [54]. If the model architecture mirrored this directly, then, (since each of the N sites can have its covalent modification present or absent independent of all the others), it would be necessary to include a species in the model to represent each combination of PTMs, leading to 2^N^ FOXO species (chemically different molecules); and, in addition, each could be present in the three compartments of the cytoplasm, nucleus or bound to DNA (transcriptionally active), making 3×2^N^ species in total. Each of these species could be present in a copy number of 0, 1, 2, … at each time point in the simulation. For the set of N = 27 modifications in Figure 2A, this would result in approximately 10^8^ species, an excessively large number, even before all the reactions to interconvert them are considered. However, simplifications can be made by grouping all PTMs that produce a similar effect on the behaviour of FOXO. This usually means all those made by a particular modifying enzyme or enzymes. So, for example, to a good approximation the three residues modified by Akt (T32, S253 and S315 in Human FOXO3) must all be phosphorylated before the effects are seen, which are to enhance export of FOXO from the nucleus to the cytoplasm [74]–[76], to change its affinity for DNA [77] and to increase its degradation rate [78]. Hence, eight combinations of phosphorylations of 3 residues can be modelled as a single collective phosphorylation, which will be referred to as type Pa (Figure 2B), either present (corresponding to all 3 individual phosphorylations) or absent (corresponding to any other combination); this reduces the resulting number of FOXO species from eight to two, i.e. by a factor of four. That all three residues must be modified is shown by the results of mutagenesis [74] and by the fact that FOXO6, which lacks one of them, is not regulated by nuclear-cytoplasmic shuttling [79]. In addition, the same three residues are modified by SGK [80] as well as Akt with similar effects; although the detailed kinetics will be different, for modelling purposes we approximate them as the same, i.e. all reactions are duplicated with Akt replaced by SGK (these two enzymes are in any case difficult to distinguish [62]) and the rates the same. Analogously, the three Akt isoforms are treated as a single Akt enzyme.

**Figure 3:**
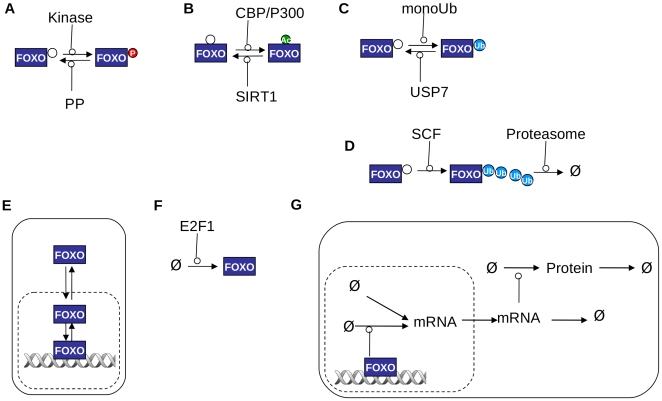
Fundamental reaction types in the model. A: phosphorylation/dephosphorylation; B: acetylation/deacetylation: C: monoubiquitination/deubiquitination; D: polyubiquitination; E: translocation between cytoplasmic, nuclear and DNA bound states; F: synthesis; G: FOXO-dependent protein synthesis.

**Figure 4:**
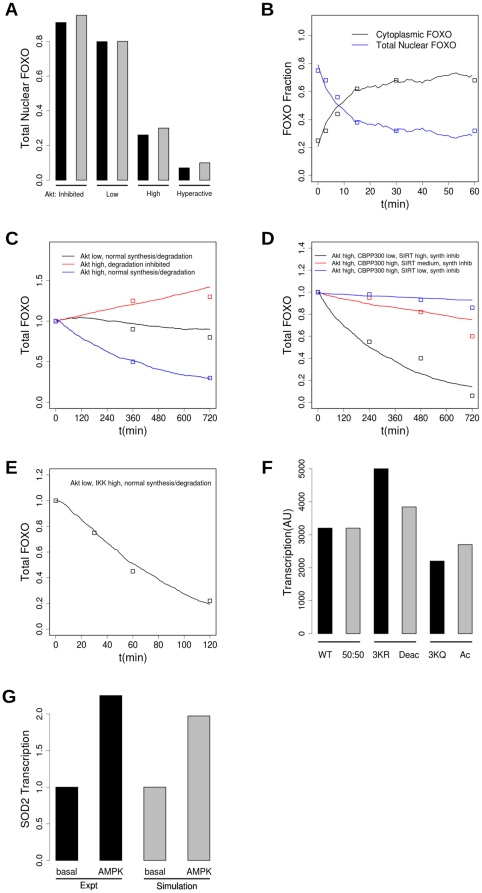
Behaviour of the model compared to experiment. A: total levels of nuclear FOXO in experiment (black) and simulation (grey) under conditions when Akt is inhibited (data from Biggs et al. [75] fig 1 D/K; CV1 cells treated with wortmannin, simulation with active Akt = 10), low (data: Biggs et al. fig 1B/I; cells serum starved, simulation: Akt = 1.75×10^3^), high (data: Biggs et al. fig 1 A/H: cells growing asynchronously in serum, simulation: Akt = 3×10^4^), and hyperactive (data: Biggs et al. fig 1F/M: constitutively active PI3Kinase, simulation Akt = 6×10^5^). B: kinetics of transport of FOXO to cytoplasm. Experiment [75]: CV1 cells are serum-starved then growth factors are provided. Simulation: pre-equilibrated for 2 h with Akt = 2×10^3^, then Akt increased to 2.5×10^4^ at t = 0. C: FOXO synthesis and degradation. Red: Expt: insulin 0.1 nM, MG132 10 µM; model: Akt = 10^5^, Proteasomes = 0. Black: Expt: serum-starved, model: Akt = 100, Proteasomes = 10^3^), blue: Expt: insulin 0.1 nM; model: Akt = 10^5^, Proteasomes = 10^3^). Experimental data taken from HepG2 cells (Matsuzaki et al 2003 [78]). D: Acetylation protects FOXO from degradation. Data points from βTC-3 cells (figure S5 in Kitamura et al [97]). Black: SIRT high (copy number 10^3^), CBPP300 low (10); Red: SIRT moderate (200), CBPP300 active (10^3^); Blue: SIRT low (10), CBPP300 high (10^3^). Synthesis is inhibited (E2F1 = 0) and Akt active (10^5^) in all simulations. E: degradation is accelerated by IKK activation: experimental data from MCF7 cells, from Hu et al [98] figure 5F, simulation: IKK = 10^5^. F: Effect of acetylation on transcription: experiment: (from Matsuzaki et al Fig 1B [99]) FOXO1- mediated transcription in HepG2 cells of 3×IRS-MLP-luc by wild-type, 3KR (acetylation-resistant) and 3KA (acetylation-mimic); simulation: nuclear SOD2 mRNA with copy number of CBPP300 = 10^3^, copy number of SIRT1 = 10^3^ (50∶50), CBPP300 = 10, SIRT1 = 10^3^ (deAc(etylated)), and CBP/P300 = 10^3^, SIRT1 = 10 (Ac(etylated)); normalized so that transcription from 50∶50 = transcription from WT. G: Upregulation of transcription by AMPK: Experiment: Greer et al figure 7Aii, transcription of GADD45 with AMPK active and WT FOXO3 or 6A mutant (AMPK-phosphorylation-resistant); simulation: AMPK = 10^5^ or AMPK = 100.

**Figure 5:**
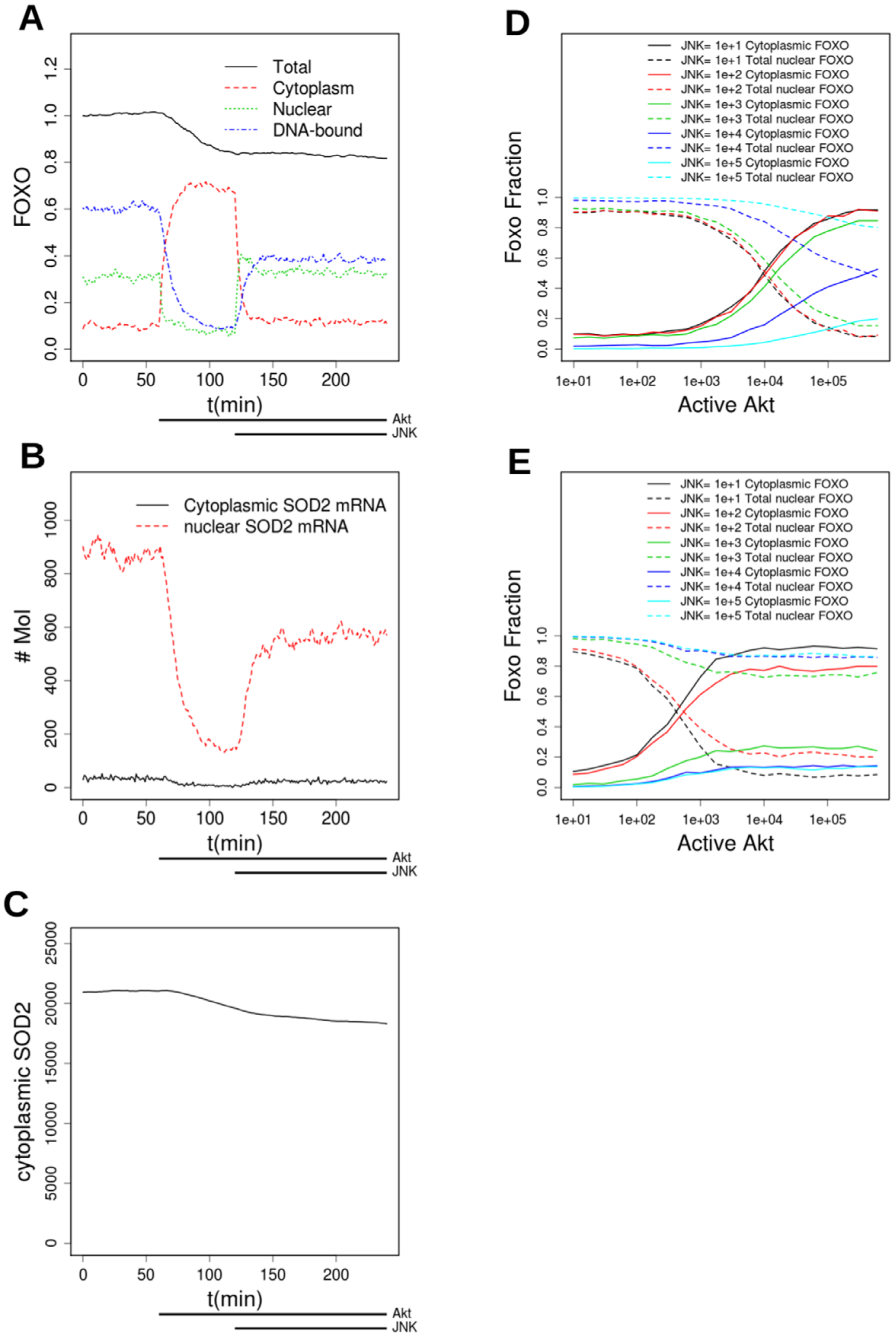
Combination of the effects of PTMs by JNK and Akt. A: FOXO is pre-equilbrated for 1440 minutes with Akt activity low (Akt = 100) and JNK inactive (JNK = 0). The PP2A level is 10^4^. At t = 60 min FOXO is set to high activity (5×10^4^); the PP2A level is 10^4^. At t = 120min the level of active JNK is also set to 5×10^4^ (times of high kinase activity shown by dosing bars below the plots). The graph shows the total amount of FOXO and its levels in the nuclear, DNA-bound and cytoplasmic compartments, as a fraction of total FOXO at t = 0.B: same experiment as A, the level of SOD2 mRNA in nuclear and cytoplasmic compartments is shown C: same experiment as A, the level of SOD2 protein is shown. D: fraction of cytoplasmic and total nuclear (nuclear+DNA-bound) FOXO, as a function of Akt copy number, for different levels of JNK. PP2A level is 10^4^. E: as for D, PP2A = 100.

Other PTMs are simplified similarly (Figure 2B). Phosphorylation by CDK2 [81] and CK1 [82], although at different residues, both lead to export to the cytoplasm and so are treated as a single modification. Five phosphorylations by AMPK [11] are considered as a single modification leading to increased transcription. IKK modifies only a single residue, so needs no simplification. The multiple modifications by JNK [83] and MST1/STK4 [45] are again combined into one, and any differences between the two enzymes are ignored. This is reasonable because both enzymes are fully and quickly activated by fairly low concentrations of H_2_O_2_ (below 100µM) (Lehtinen Figure 1A, Essers Figure 1A); they then phosphorylate FOXO within 30 mins (Lehtinen Figure 3A, Essers Figure 3A), which results in its nuclear localization (Lehtinen Figure 4EF, Essers Figure 4A) and transcriptional upregulation (Lehtinen Figure 5A, Essers Figure 4B). Four acetylations [84] are combined into one modification, as are two monubiquitinations [85]. Unlike the other PTMs, it is possible that there could be competition between the processes of acetylation and monoubiquitination, since both PTMs are attached to Lys. However, the two modifications are treated as independent in the model. Polyubiquitination, leading to degradation, is treated as a one-step process, although in fact a polyubiquitin chain must be built up stepwise; this can be modelled in detail [86] but this degree of sophistication was not included in the current FOXO model. With these simplifications, there are effectively only a maximum of 8 modifiers and 3 locations to consider, leading to a maximum of 768 chemical species of FOXO if all PTM-addition reactions are active. It is also of interest to know the total amount of FOXO having a particular PTM, since this typically corresponds to what can be measured via e.g. immunoprecipitation or a Western blot; this is the sum of the copy numbers of all the species that have that modification present, irrespective of the other PTMs that they may contain. Therefore counter variables are also introduced in the model, which are not involved in any reactions but simply sum appropriate subsets of the FOXO species.

We now turn to the second problem, that of addressing the kinetics of the reactions in which these species are involved. First, consider the reactions involved (Figure 3). A particular FOXO species may be phosphorylated by any of the five types of kinase in Figure 2B and desphosphorylated by the corresponding phosphatase (Figure 3A). In this model, only a single phosphatase is included that may catalyse any dephosphorylation reaction. FOXO may also be acetylated by the CBP/P300 enzyme and deacetylated by SIRT1 (Figure 3B), monoubiquitinated and de-ubiquitinated by USP7 (Figure 3C), polyubiquitinated by the SCF complex (or MDM2) and the resulting polyubiquitinated form degraded (Figure 3D), translocated between cytoplasm, nucleus and a DNA-bound state (Figure 3E), and synthesised (Figure 3F). It may not be necessary to consider all these reactions as being active simultaneously (indeed in this paper we do not consider more than two or three different reactions being active at any time), but the structure of the model allows them all to be so. When the FOXO is bound to DNA, it stimulates the production of the mRNA for each gene that it regulates; we assume that there is also a “basal” production of this mRNA, a translocation of the mRNA to the cytoplasm, where it may be degraded or translated to protein, which may itself be degraded. Only in its own synthesis reaction does the FOXO represent a single species in the model, since it is synthesised in the cytoplasmic compartment without any PTMs; otherwise it may already have any combination of the PTMs applied by any of the other reactions, and may also be present in any compartment. Hence, up to several several thousand reactions interconverting FOXO species must be taken into consideration. However, the reactions change only one of the PTMs at a given step, i.e. each FOXO species can be converted into only nine others. These pre-existing modifications will, however, in general affect the rate constants for the forward and backward steps. Usually, the way that multiple modifications interact has not been measured in detail. In the model described in this paper, the process has been treated simply by multiplying the effects that each of the PTMs has in isolation on the relevant rates. These effects are therefore modelled as parameters, which will be referred to as *Multiplicative Factors* (MFs) subsequently, that multiply the “basal” rate, For example, for a species with both Akt and JNK phosphorylations, the effect on translocation is the basal rate×MF due to Akt×MF due to JNK. This is clearly likely to be a fairly gross approximation, but it is necessary to enable the model to be parameterized; its justification will thus be in the empirical validity and usefulness of the model. Of course, as most detailed measurements are made, it will be possible to modify the model to incorporate them.

For the transcription reactions represented in Figure 3G, the number of reactions in the model would also be multiplied by the number of FOXO-regulated genes. Currently, though, transcription is considered to be that of only two genes, SOD2 (MnSOD) and InsR, with identical kinetics. Transcription results are shown only for SOD2.

For many of the regulatory reactions included in the model, there are not explicit time courses, or only a few points. They are modelled by mass-action kinetic equations of the following form:
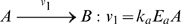


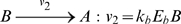


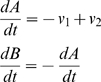
In these equations, *A* and *B* represent FOXO species with different PTMs, and the forward and back reactions occur at rates *v_1_* and *v_2_*, catalysed by enzymes *E_a_* and *E_b_*. The experimental information often amounts to a knowledge of *(A/B)_eq_* and some idea of the relaxation time, from an experiment when one of *A* and *B* is initially at very low concentration, then the enzyme that produces it is activated. From the rate equations, first with the time derivative equal to zero, and second with one of *A* or *B* (near)-zero, it is simple to see that this determines pseudo-first order rate constants, *k′_a_ = k_a_E_a_* and *k′_b_ = k_b_E_b_*. Then, we choose appropriate values for the copy number of *E_a_* and *E_b_* in the range of 10^4^ to 10^5^ when fully activated (10^5^ is typical of Akt when stimulated by insulin [87]); and set the second order rate constants *k_a_* and *k_b_* accordingly, as shown in Table 1.

**Table 1:** Parameters of the model (FOXO synthesis/degradation and PTM addition/removal processes).

process	2° rate constant (#min^−1^)	Enzyme	Enz #	1° rate const (min^−1^)	PTM modify-ing rate	Rate MF	Reference
Synthesis (NULL→FOXO)	0.0055	E2F1	100	5.5			[78] Figure 1
Degradation (FOXO_pUb→NULL)	1×10^−4^	Protea-some	10^3^	0.1			“
FOXO→FOXO_Pa	5×10^−5^	Akt	10^4^	0.5			[27] Figure 1C
FOXO_Pa→FOXO	5×10^−5^	PP2A	10^4^	0.5			“
FOXO→FOXO_Pb	5×10^−5^	CDK2	10^4^	0.5			[81], [82]
FOXO_Pb→FOXO	5×10^−5^	PP2A	10^4^	0.5			“
FOXO→FOXO_Pc	5×10^−5^	AMPK	10^4^	0.5			[11]
FOXO_Pc→FOXO	5×10^−5^	PP2A	10^4^	0.5			“
FOXO→FOXO_Pd	5×10^−5^	IKK	10^4^	0.5			[98]
FOXO_Pd→FOXO	5×10^−5^	PP2A	10^4^	0.5			“
FOXO→FOXO_Pe	5×10^−5^	JNK	10^4^	0.5			[83]
FOXO_Pe→FOXO	5×10^−5^	PP2A	10^4^	0.5			“
FOXO→FOXO_Ac	1×10^−4^	CBP	10^3^	0.1			[96], [99]
FOXO_Ac→FOXO	1×10^−4^	SIRT	10^3^	0.1			“
FOXO→FOXO_mUb	2×10^−4^	E3	10^3^	0.2			[85]
FOXO_mUb→FOXO	2×10^−4^	USP7	10^3^	0.2			“
FOXO→FOXO_pUb	1×10^−6^	SCF/MDM2	10^3^	10^−3^			[78] Figure 1; also [94]
					Akt-Phos Pa	3	[78] Figure 1
					IKK-Phos Pd	6	[98] Figure 5F
					Acetyl Ac	0.033	[97] Figure S5

Rate constants of FOXO synthesis/degradation and PTM addition/removal processes. For a given process, the first-order rate constant is given, and, for enzyme catalysed reactions, a derived second-order constant and the putative enzyme molecule number that produced it. Only the product of these, the first order constant, is related to experimental measurements. Where appropriate, a list is then given of a series of multiplicative factors (MFs) which may modify the rate for the process, depending on the presence of a another particular PTM in the FOXO species.

Transport processes are modelled with first-order kinetics:
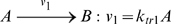


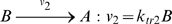
Where *A* and *B* represent FOXO species in different compartments, and the first order rate constants *k_tr1_* and *k_tr2_* can be found from the relaxation kinetics. The rate constants for transport processes are given in Table 2.

**Table 2:** Parameters of the model (transport of FOXO and processes involving FOXO-regulated genes).

process	2° rate constant (#min^−1^)	Enzyme	Enz #	1° rate const (min^−1^)	PTM modify-ing rate	Rate MF	Reference
Cytoplasm→nuclear transport	-	-	-	0.1/0.55 = 0.182			[74], [75]
					Akt-Phos Pa	0.1	[75]
					CDK2-Phos Pb	0.5	[81] Figure 3A
					IKK-Phos Pd	0.5	[98] Figure 1
					JNK-Phos Pe	10	[83]
					mUb	10	[85]
Nuclear→cytoplasm transport	-	-	-	0.1×0.55 = 0.055			[74], [75]
					Akt-Phos Pa	10	[75]
					CDK2-Phos Pb	2	[81]
					IKK-phos Pd	10	[98]
					JNK-Phos Pe	0.1	[83]
					mUb	0.1	[85]
Nuclear→DNAbound transport	-	-	-	0.25			[76]
					Akt-Phos Pa	0.5	[77] Figure 6B
					Acetyl Ac	0.5	[99] Figure 1D
DNAbound→Nuclear transport	-	-	-	0.25×0.5 = 0.125			[76]
FOXO- simulated Transcription k_transcr_	0.3	FOXO	#(DNAbound FOXO)				[19]
					CDK2- Phos Pb	0.1	[81]
					AMPK-Phos Pc	2.0	[11] Figure 7A (ii)
Basal transcription k_basalt_	-	-	-	3			[100]
RNA export k_exp_	-	-	-	0.22			[100]
RNA translation k_transl_	-	-	-	1.23			[100]
RNA degradation k_mdeg_	-	-	-	5.622			[100]
Protein degradation k_pdeg_	-	-	-	1.9×10^−3^			[100]

Rate constants of FOXO translocation reactions and transcription, translation and degradation processes of FOXO-regulated genes/proteins. The first-order rate constant is given for these processes, except for basal transcription, which is zero-order. Where appropriate, a list is then given of a series of multiplicative factors (MFs) which may modify the rate for the process, depending on the presence of a particular PTM in the FOXO species.

We have assumed that at the levels of “high active kinase” (10^4^ molecules or more) the rates are high (half-life of a few minutes) compared to processes like the relocation/degradation/transcriptional output of FOXO. This is true of Akt-phosphorylation [27] and is typical of phosphorylation processes, (though perhaps less so of acetylation).

For the transcriptional outputs, the relevant reactions are
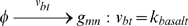


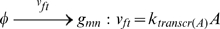


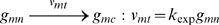


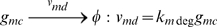


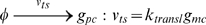


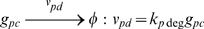
Where *φ* is null, *g_mn_* is the mRNA in the nucleus of gene *g* (currently either SOD2 or InsR), *g_mc_* is the cytoplasmic mRNA of gene *g*, *g_pc_* is the cytoplasmic protein corresponding to gene *g*, and *A* is a DNA-bound FOXO species. The first- or zero-order rate constants are given in Table 2.

A python script was written to generate the species and reactions: this script is available as supplementary dataset S2. To comply with modelling standards and simplify exchange and re-use, SBML [88] is used to represent the model; but, as a further simplification, rather than generating SBML directly, the python script produces output in SBML-shorthand [89], a more compressed and human-readable format which nevertheless can be converted without loss of information to SBML.

Simulations were carried out using the gillespie2 stochastic simulator in stand-alone mode and also through BASIS, which provides a web interface to gillespie2 and a database to store the results [73], [90]. Analysis was also carried out using R. Sensitivity analysis was performed with COPASI [91].

As we have said above, the kind of equations relating to fractional locations or degrees of up/down regulation do not permit the number of molecules involved to be known exactly. The initial number of FOXO molecules of all species was set to be 1000. This is probably an underestimate, but is the approximate number of consensus FOXO binding sites, GTAAACAA [92], within 1 kbase of the complete set of transcription start sites in the genomes of the mouse and fly. This number is large enough that the difference between a stochastic and deterministic simulation is often unimportant; however, in this case, the large number of different FOXO species means that the copy number of each species can still be low – a situation that will arise commonly if multiple regulations are included in a model [93]. In addition, the copy number of mRNA species for the transcribed gene is low. For both these reasons a stochastic representation is more appropriate than a deterministic.

## Results

### Fitting the Model to Experimental Data

The first regulation of FOXO to be discovered was its Akt-phosphorylation and translocation between nucleus and cytoplasm. Simply stated, in the absence of Akt-activation, FOXO is primarily nuclear, while activation of Akt, usually by the insulin-signalling pathway, leads to sequestration of FOXO in the cytoplasm. Experimentally, FOXO localization is usually measured as the fraction of cells having FOXO mostly nuclear, mostly cytoplasmic or both nuclear and cytoplasmic, as determined with fluorescent-labelled FOXO. In the current model, the fraction of FOXO in the nuclear or cytoplasmic compartment will be treated as equivalent to the experimentally determined fraction of *cells* having FOXO mostly nuclear or cytoplasmic. Those cells which are experimentally observed to have FOXO both nuclear and cytoplasmic are counted together with those where it is wholly cytoplasmic (an equal division between nuclear and cytoplasmic was also considered, but the assignment to cytoplasm gives a better fit). In the model the total nuclear FOXO is simply the sum of that in the nuclear and DNA-bound compartments. The basal rate of redistribution is ∼0.1 min^−1^, to give a half-life of the order of 10 minutes, with the cytoplasmic-nuclear rate slightly higher, and the nuclear-cytoplasmic lower, for unmodified FOXO, to give an equilibrium favouring the nuclear form. The basal rates of DNA binding and unbinding are chosen so that, of the total nuclear FOXO, about two-thirds is DNA-bound and one-third nuclear but not bound to DNA.

In Figure 4A we show the generally good agreement between the total nuclear FOXO fraction in experiments in CV1 cells (Figure 1A–N [75]) and simulation, under gradually increasing levels of Akt activation, corresponding in experiment to inhibition of PI3K with wortmannin, serum-starvation, provision of growth medium, and constitutive activation of PI3K. (PI3K is a kinase upstream of Akt in the IIS pathway).

In Figure 4B a timecourse of the redistribution of FOXO from nuclear to cytoplasmic upon Akt activation is shown. In the experiment (Biggs et al. Figure 1O [75]), CV1 cells are serum starved for 2h and then stimulated for 1h with IGF-1. Using the model, the best fit to this is obtained with active Akt of 2×10^3^ in the pre-equilibration level, which is then increased to 2.5×10^4^ to correspond to the IGF stimulation. The experiment lags slightly behind the simulation result at around 5 or 10 minutes, but this is reasonable given that that in the simulation the level of active Akt is set directly whereas in the experiment it must be activated through the IIS pathway, with a delay of the order of a few minutes.

Now let us consider the synthesis and degradation of FOXO. This was first studied in detail in experiments on HepG2 cells by Matsuzaki et al [78]. These experimental results, together with model output, are shown in Figure 4C. For synthesis, it has been shown that, if the proteasome is inhibited with MG132, the amount of FOXO1 increases in the cell by 20% in 6h. Taking the equilibrium copy number of FOXO of all chemical species to be 1000, the rate of increase of FOXO is then approximately 5.5 molecules min^−1^, shown in Table 1 as a pseudo-first order rate constant. Breaking this down in to a second-order constant × the copy number of the E2F1 transcription factor, and choosing this copy number = 100, gives the basal value of the rate constant 0.0055 min^−1^. Then, without proteasome inhibition, the amount of FOXO in the cell was quantified with and without insulin stimulation, i.e. with and without Akt activation, over 12h. Although proteasomes are present in the nucleus, it has been convincingly shown that degradation of FOXO occurs only in the cytoplasm [78], [94]. It has also been shown that phosphorylated FOXO is degraded faster. Assuming the basal synthesis level is maintained subsequently, we simultaneously fitted the pseudo first-order polyubiquitination and degradation rates and the MF multiplying the rate at which Akt-phosphorylated FOXO is degraded compared to unphosphorylated. The best fit was obtained with first order rate constants of 10^−3^ min^−1^ for polyubiquitination, 0.1 min^−1^ for degradation, and an MF of 3. Taking the copy numbers of the modifiers to be 10^3^ for both the total of ubiquitin ligases (SCF+MDM2) and the proteasome, this gives second order rate constants of 10^−6^ and 10^−4^ min^−1^. With these parameters, Figure 4C shows that, when degradation is inhibited, the amount of FOXO in the cell increases despite the activity of Akt (data in red), whereas, without inhibition of degradation, FOXO molecule number decays very slowly when Akt activity is low (data in black), or with a half-life of about 6h when Akt activity is high (data in blue).

Other PTMs significantly modulate the degradation of FOXO. Acetylation is one of these, and is of particular interest because of the suggestion that the FOXO- and histone- deacetylase, SIRT1, may also modulate organism lifepan. The timescale of both acetylation and deacetylation is of the order of 10 minutes when highly activated. This is shown in van der Horst 2004 [95] Figure 4, in response to high concentrations of H_2_O_2_, 200 and 500 µM, that activate CBP/P300 and fully acetylate FOXO4 in 30 minutes (although at lower concentrations, such as 20 µM, acetylation is still low after 1h but is more or less complete after 4h). Hence, it is reasonable to choose pseudo-first-order rate constants of ∼0.1 min^−1^, and we make the choice of the copy number of CBP/P300 and SIRT1 to be 10^3^ when fully activated, and a second order rate constant of 10^−4^ for each reaction. SIRT1 and CBP/P300 are mostly nuclear [96], so in the model these reactions do not occur in the cytoplasmic compartment. Regarding its interaction with degradation, experiments by Kitamura et al [97] on FOXO1 in betaTC3 cells (pancreatic cells, in which Akt is permanently activated by endogenous insulin) show that acetylation exerts a strong protective effect against degradation. This is shown in Figure 4D, where model output is fitted to experimental data. This fit is obtained with acetylation reducing the rate of polyubiquitination by a factor of 30, and SIRT and CBP/P300 levels that give high, medium and low levels of acetylation.

Another PTM that affects degradation, this time accelerating it, is phosphorylation by IKK. Again, phosphorylation is chosen to be a rapid process in comparison with degradation. The fit in Figure 4E is produced by a saturating level of IKK activation, with a MF of 6 for polyubiquitination of IKK-phosphorylated FOXO. IKK phosphorylation also leads to FOXO translocation to the cytoplasm even if Akt is low. This leads to good agreement with experimental data from Hu et al on FOXO3 [98].

Acetylation also affects transcription, as shown in Figure 4F, where experimental measurements [99] of transcription by wild-type FOXO1, an acetylation-resistant 3KR mutant and an acetylation-mimicking 3KA mutant are compared with transcriptional output of the model with SIRT and CBP/P300 levels that produce 50% of FOXO acetylated, mostly deacetylated, and mostly acetylated. The DNA-binding of the acetylated form is decreased by a factor of two, as was measured directly [99]. The agreement is reasonable, especially considering that the mutants may in any case not behave identically to the acetylated or deacetylated forms.

FOXO may also be modified by AMPK, with modulation of transcription but not subcellular localization [11]. Again, we assume that AMPK-phosphorylation is a rapid process, and when fully phosphorylated, the FOXO-stimulated transcription rate is increased by a factor of two. As shown in Figure 4G, this is in good agreement with the experimental data for transcription from several genes including GADD45, though the experimental result seems to be gene dependent, a level of complication not included in the model currently. In Figure 4G, experimental data for transcription of GADD45 by WT FOXO3 with AMPK activated, and transcription under the same conditions from a 6A mutant of FOXO that is not phosphorylated by AMPK, is compared with simulation data with AMPK fully active (copy number 10^5^) and mostly deactivated (copy number 100).

JNK-phosphorylation broadly opposes the effects of Akt-phosphorylation, and is discussed in the subsection on scenarios of multiple FOXO regulation below. Other modifications such as CDK2-phosphorylation and monoubiquitination have similar effects, causing alteration of the nuclear-cytoplasmic equilibrium and FOXO-mediated transcription. They can be modelled similarly (data not shown).

Several parameters of the model relate to transcription of mRNA, its transport to cytoplasm, translation to protein and and degradation; and also to the degradation of the protein. Lacking absolute quantification of protein or mRNA level, or measurement of mRNA or protein lifetime, we have used generic values [100]. SOD2 protein level seems to be upregulated to about the same degree as mRNA on a timescale of 1 day [19], consistent with a protein half-life of up to about 8 hours. Only the transcription rate depends explicitly, in certain cases, on the PTM state of FOXO. In the future these parameters could be made gene-specific as required. Example models (in SMBL format) producing curves in Figure 4C–E are given as supplementary datasets S3, S5 and S7. These datasets, together with all other supporting information, are also available in the single dataset S9.

### Sensitivity

We have investigated the sensitivity of the concentrations of the species of the model as a function of the parameters, using COPASI [91], in deterministic simulations of the models used in the fitting described above. The sensitivity was calculated at a certain time point, particular to each simulation, where initial transients had died away (most of the enzyme-catalysed PTM adding/removing reactions had come into pseudo-equilibrium, while the concentration of SOD2 protein, which has a longer timescale, was near a stationary point). It was not appropriate to find the long-time equilibrium as in several of the scenarios simulated the model did not have a non-trivial steady state. Scaled sensitivities, i.e. *(k/X) dX/dk* where *X* is a concentration and *k* a parameter, were considered. Examples of the detailed results are available as supplementary Datasets S4 (corresponding closely to the model S3), S6 (corresponding to S5) and S8 (corresponding to the model S7). The most important parameters depend, of course, on the regime that the model is in (which PTMs are active, etc): sensitivities are low to those parameters which only affect reactions in which the species involved are present at low concentrations. In general, synthesis/Akt-phosphorylation and polyubiquitination (but not degradation itself) and transport processes, especially between nucleus and cytoplasm, tend to affect the FOXO concentrations and the concentrations of the output gene, with high sensitivities (of magnitude up to 1). Only a few FOXO concentrations, and not the output gene concentrations, have a high direct sensitivity to reactions involving acetylation/deacetylation. We remark that, although the results are sensitive to Akt-phosphorylation and dephosphorylation, they are not sensitive to an increase or decrease of both by the same factor, because these reactions (and others of PTM addition and removal) occur on a shorter timescale than other processes in the model such as FOXO synthesis and degradation. Datasets S4, S6 and S8, together with all other supporting information, are also available in the single dataset S9.

### Scenarios of Multiple FOXO Regulation

Having shown that experimentally-determined responses to single and some multiple regulations can be reproduced by the model, we now move on to study the behaviour of the model in scenarios involving multiple regulations. A simple combination of effects is shown in Figure 5A–C. A simulation has been pre-equilibrated for 1 day, to allow the SOD2 protein to reach equilibrium. Akt activation (starting at t = 60 min and remaining active for the rest of the simulation) causes FOXO translocation to the cytoplasm; accordingly, transcription almost stops and the cytoplasmic and nuclear concentrations of mRNA fall; the level of the protein also begins to fall, although the effect is not very large on a 1 hour timescale. After a further 60 minutes, JNK is activated, and in large measure overrides the effect of Akt and causes the FOXO to translocate back from the cytoplasm to the nucleus. Once in the nucleus, however, it will be observed that the ratio DNA-bound/nuclear is lower than before, and the FOXO level is also slightly lower as some has been degraded while it was cytoplasmic. Accordingly, although the mRNA level recovers somewhat, it is not to its previous level, and the protein continues to fall, though very slowly. In this simulation, the level of both kinases is set to 10^5^ when they are active. The PP2A level is 10^4^ molecules, so the PTM that the kinases confer is present in about 80% of the FOXO molecules.

Experiments in which JNK is activated by H_2_O_2_ show an interplay between the opposing effects of JNK and Akt; JNK tends to override Akt (as in A–C) but if Akt activity is particularly high it may override a low level of JNK activation [83]. Detailed dose-response data that would enable a full parameterization is not available, but Figure 5D and E show that the model can capture these effects. As Akt increases it tends to drive FOXO to the cytoplasm, but the effect can be reversed by JNK. It is interesting to note the effect of reducing the activity of protein phosphatases: in D the PP2A level is high (copy number 10^4^), while in E it is much lower. It is noteworthy that, with PP2A low and without JNK, a lower level of Akt activation (in E compared to D) is sufficient to drive FOXO to the cytoplasm, but also a lower level of JNK activity suffices to retain it in the nucleus even at a high level of Akt activation (compare the response to JNK = 10^3^). That PP2A, JNK and Akt could all change together is a likely scenario, because oxidative stress tends to inactivate protein phosphatases due to their reliance on a catalytic cysteine that contains an easily oxidised SH group.

**Figure 6:**
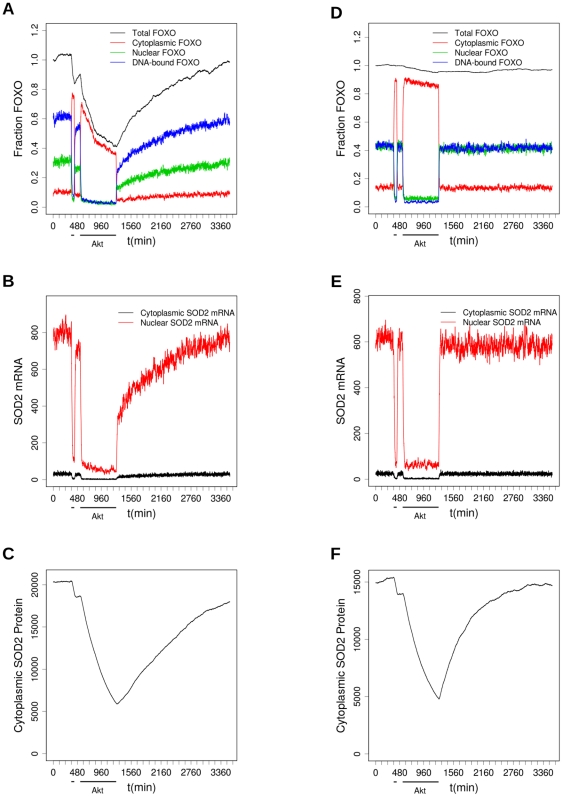
The effect of brief and prolonged Akt activity interacting with degradation and acetylation. Before the start of the time course in the figure, FOXO is pre-equilibrated for 2520 minutes to allow its target gene SOD2 to come into equilibrium, with Akt very weakly active (copy number 100). A–C shows a simulation where acetylation is low (SIRT1 = 10^3^, CBP/P300 = 10), whereas in D–E CBP/P300 is high (SIRT1 = 10, CBP/P300 = 10^3^) so that FOXO is 99% acetylated. As shown by the dosing bars, Akt is activated at t = 360 min for 60 min, and again at t = 540 min for 720 min. The panels show: (A,D): total FOXO and the amount in each compartment, as a fraction of the total at t = 0; (B, E) mRNA in nucleus and cytoplasm for the output gene SOD2; (C,F) SOD2 protein level.

Another example is shown in Figure 6, which shows the effect of a short and long stimulation by Akt under different background PTM states. This *in-silico* experiment was suggested by comments made in Calnan and Brunet [54]; to our knowledge it has not been experimentally investigated, so the simulation results constitute an experimentally verifiable prediction made by the model.

First, consider the effects of short and long-term activation of Akt alone, (Figure 6A–C). The system was pre-equilibrated at low Akt for 2880 minutes (2 days) before the first period of Akt activation. The last 360 minutes of this is shown in Figure 6, so that the mainly nuclear (DNA-bound) location of FOXO is apparent. At t = 360, Akt is activated for 60 minutes (set by an event from 100 molecules to 10^5^). This initial burst of Akt activation causes FOXO translocation to the cytoplasm and transcription almost stops (Figure 6B). However, once active Akt is reset to a low level (back to 100 molecules t = 420 for the next 120 min) the FOXO rapidly relocates to the nucleus and transcription resumes at almost the same level it had initially. A second, longer period of Akt activation then follows (10^5^ active Akt molecules from t = 540 for the next 720 min (12 hours) ), again causing translocation of FOXO and almost halting transcription of FOXO target genes. This pulse is long enough for the level of FOXO to decline by more than half in the course of it, as a result of polyubiquitination and resultant degradation of the cytoplasmic FOXO. Hence, when the second pulse of high Akt activity ends (at t = 1260) and FOXO returns to the nucleus, the transcription level (Figure 6B) is at first also less than half what it was before the Akt pulse. The simulation is continued for a further 1 ½ days (until t = 3420), during which the transcription level recovers, as FOXO is re-synthesised, though even at the end of this period it has only just regained its initial level. Consequently, the level of SOD2 protein (with a half-life of 8h) also declines appreciably during the long Akt pulse, and recovers only slowly thereafter. In this simulation the copy number of CBP/P300 was set to 10 molecules and SIRT1 to 1000, levels designed to ensure that acetylation is slight, and the levels of other modifying enzymes were set to zero.

The same protocol of brief and prolonged Akt activity has a different outcome if the FOXO is more strongly acetylated (Figure 6 D–F), an effect which may be produced either by increasing the level of active CBP/P300 and/or decreasing the level of SIRT1; in this simulation the levels were CBP/P300 = 1000, SIRT1 = 100 throughout the pre-equilibration period and the timecourse shown, leading to 90% acetylation of FOXO. E2F1 was reduced to ensure that the copy number of FOXO was about the same in A and D. Acetylated FOXO binds DNA less strongly, but it is also stabilized against polyubiquitin-mediated degradation. As a consequence, the basal level of FOXO mediated transcription is lower (only 75% of its level when FOXO is unacetylated; Figure 6B vs Figure 6E) but almost none of the FOXO is degraded during the long Akt pulse, so that when the second pulse of Akt activity ends, transcription recovers almost immediately to its basal level in this PTM background. Hence, although equilibrium level of SOD2 is also lower, and it is degraded as before during the long Akt pulse when FOXO is sent to the cytoplasm, it recovers more quickly to its initial level (Figure 6F cf Figure 6C).

This demonstrates how the model is capable of making experimentally verifiable predictions of the effects of PTM-inducing treatments applied according to various time-dependent protocols.

## Discussion

The model presented here provides a significant first step towards a detailed model coupling ageing-related stress and nutrient response pathways with the stress resistance. It also provides an extensible modelling framework into which future information could be integrated. Although complex, because of the need to treat multiple chemical species, we believe that there is no other approach that can integrate information from multiple regulatory pathways into a single framework. We have shown that, even without detailed up- and downstream pathways, it is capable of reproducing experimental data on many features of FOXO regulation. Moreover, in Figures 5 and particularly 6, we have shown how the multiple regulations of FOXO alone can produce complex spatio-temporal dynamics, such as the faster recovery of antioxidant levels after the end of a long period when FOXO acetylation is at a high level.

Despite the large amount of work done on FOXO transcription factors, it is not easy to parameterize the model; time courses are generally not available, and different organisms and different tissues are widely used, so we have frequently had to argue by homology between different tissues or organisms, though human FOXO1 was the paralogue most often used and cell lines such as CV1, HepG2, βTC-3 and MCF7 were used. Hence the fact that we have referred to the species in the model as FOXO rather than FOXO1 or some other specific paralogue.

The modifying processes have been represented by simple 2° mass-action rate laws. This is a consequence of two factors: first, there are few quantitative timecourses available, but more usually a series of Western blots (sometimes only showing the presence or absence of a treatment and an indication of the time over which the change occurs). With this data, the forward and backward rates of a first order process and its inverse can be estimated, but the multiple constants of more complicated kinetics cannot. Secondly, the simplification of combining multiple phosphorylations into a single collective modification leads naturally to simple kinetics where the FOXO species appears linearly in the rate expression; nevertheless it is likely that more detailed timecourses would show that the kinetics do not follow this kind of behaviour. For example, with multiple regulations, it is to be expected that the fully phosphorylated form will appear as a concave-upwards function of time, such as a cubic function when three phosphorylations occur, in response to a step increase of the enzyme producing it, rather than the linear timecourse of the 1° model. This may often seem to correspond to an apparent initial delay in experiment. As detailed time course data becomes available it may, therefore, be necessary to revise this assumption.

Transport into and out of the nucleus has also been represented by simple first order processes with rates dependent on the PTM state, when in fact the process is more complex, involving binding of the FOXO to two 14-3-3 proteins, masking the Nuclear Localization Signal, and its resultant export to the cytoplasm [62]. This export from the nucleus is itself a multi-step process, involving binding to the nuclear pore complex and several other cofactors. Again, this may alter the kinetics in some regimes. It is interesting to note that the action of JNK in causing nuclear localization occurs through multiple mechanisms: JNK phosphorylates both 14-3-3 and FOXO, causing them to unbind from each other in the cytoplasm, and also attenuates Akt activation by phosphorylating IRS. The action seems to be evolutionarily conserved, but the JNK-phosphorylation sites are conserved much more highly in 14-3-3 than FOXO [63], so the effect is probably a result of the 14-3-3 modifications. The model, in which 14-3-3 is not explicitly represented, can capture this simply as a nuclear re-localization response which is conserved even if the JNK-phosphosites are not (cf. Figures 1A/2A).

The negative second derivative of the experimental curve of FOXO molecule number against time (Figure 4C) suggests that there is negative feedback inhibition of FOXO synthesis, though this is not included in the current model. This may also explain why acetylation does not seem to produce a large increase in FOXO molecule number.

In general, the kinetics of the phosphatase-catalysed reactions are much less certain that those of the kinase-catalysed reactions. Most of the time they have not been studied in detail. Hence, we have assumed a single “general phosphatase”, assumed to be PP2A, to reverse all the kinase-mediated phosphorylations in the model.

When the localization of FOXO alters experimentally in response to changes in PTM, it is experimentally observed that there is a change in the fraction of cells in the population having FOXO primarily nuclear, primarily cytoplasmic, or present in both compartments (see for example Figure 3 of Brunet 1999 [74], Figure 1 of Biggs et al. 1999 [75] or Figure 4 of Essers et al. 2004 [83]). Although the stochastic modelling carried out here means that there is continuous fluctuation in the amount of FOXO in the various compartments of the model (cytoplasm, nuclear and DNA-bound), we found that the fluctuation is usually small, about 10 molecules, i.e. 1% of the total amount of FOXO, and the average number of FOXO molecules in each compartment varies smoothly as the PTM level alters. This seems somewhat different to the behaviour reported about cell cultures; however it is likely that there is in fact a continuous variation in the amount of FOXO in nuclear and cytoplasmic compartments, but the impossibility of quantifying this amount means that the simple three-state classification must be adopted, and all cells where FOXO is present in more than small amounts in both compartments will be classified as having FOXO nuclear+cytoplasmic, whether the ratio be 0.2∶0.8 or 0.8∶0.2. Turning to the question of the differences between cells in a population, it might at first be thought that the experimentally observed behaviour corresponds to stochastic fluctuations in the response of particular FOXO molecules. However, since in the model these fluctuations were very much smaller, and moreover since it appears from the published data that the FOXO localization in a particular cell is more or less stable in time, we believe this is unlikely to be the case. It could instead be a consequence of differences between cells in the expression level of upstream components of the insulin signalling pathway. In future, heterogeneity in a population of models could be studied, and/or alternative model architectures, such as positive feedbacks, could be investigated to try to increase the observed fluctuations to obtain behaviour closer to that seen experimentally. These are currently beyond the model boundaries.

Several details of the reactions which could potentially affect the model's behaviour have been omitted. For example, cofactors essential for the formation of PTMs, such as ATP, ubiquitin, Acetyl-CoA and NAD+, are not explicitly included in the model. We also remark that, although the model reproduces the experimental result that acetylation downregulates FOXO-mediated transcription, and deacetylation upregulates it (Figure 4F), the situation experimentally is more complex than this suggests, with transcription found to vary in a gene-dependent way, some genes being downregulated and some upregulated [96], [99], [101]; reviewed in [102]. A plausible explanation for this is given by Daitoku et al [84], whose work showed that histone acetylation and deacetylation is carried out, with an effect on transcription, by the same enzyme pair that modifies FOXO. This could be handled by extending the model to include acetylated and deacetylated states for the FOXO-regulated genes as well as for FOXO itself. Transcription downstream of FOXO is not the focus of this paper and has been limited to two genes. However, the model could be easily extended to include multiple genes, with gene-specific values of the basal and FOXO-stimulated transcription rate.

In addition to its direct action as a DNA-binding transcription factor, FOXO may bind to other TFs and modulate their activity. This occurs for example with nuclear receptors such as HNF-4 [103] and ER (Estrogen receptor) [104], [105], as well as PR, GR, RAR and TR [104]. FOXO seems usually to reduce the transactivation function of the other TF but may also increase it, as the case of RAR and TR. In a similar way, FOXO may also bind to another TF acting as a transcriptional repressor and relieve this repression, as seems to happen with p53 repression of SIRT1 transcription [29] or Cs1 repression of Hes1 [106]. Behaviour of this kind could be treated by extending the model to introduce reactions in which FOXO species, probably that subset which is nuclear but not DNA-bound, bind to other TFs to form species representing the FOXO-TF complexes. Depending on the other TF and the gene, the resulting complexes themselves could be assigned low or no transcriptional activity, corresponding to FOXO acting as a transcriptional repressor, or a higher transcriptional activity than the other TF alone, corresponding to FOXO acting as a transcriptional co-activator. It was found in Hirota et al. [103] that the binding between FOXO and HNF-4 itself depends on the phosphorylation state of FOXO; this could be treated very naturally in the modelling framework used here. The cost in increased complexity of the model depends on the other TF. If it can be treated simply the cost would be similar to that of adding an extra class of PTM to FOXO, but if it has many states itself (representing different localizations, different PTMs etc) then the number of species representing complexes is the product of the number of FOXO species and the number of species of the other TF, and care would need to be taken to keep this manageable. Similar considerations arise if the number of regulated genes in the model is also high.

Even the large number of regulations considered in this paper does not exhaust FOXO's repertoire. A potentially important omission in the current model is that of additional stress response phosphorylations made by p38 MAPK (MAPK14) and ERK (MAPK1), which may not be synonymous in their effect to those by JNK. It has been shown that ERK and p38 MAPK modify mouse FOXO1 [107], and the p38 MAPK orthologues pmk1-3 modify daf-16 in *C elegans* [108]. At least in worms, the effect of modification by p38 is similar to that by JNK, i.e. translocation of FOXO to the nucleus; however there is some evidence that ERK in mammals has a different effect, causing increased MDM2-mediated degradation and possibly translocation from nucleus to cytoplasm (although inhibition of MEK, upstream of ERK, fails to reverse this [109]). At the cost of further increasing the number of species in the model, these effects could be included by adding additional PTM states within the framework of the existing model and choosing appropriate rate constants to reproduce experimental data as far as possible. FOXO can also be methylated [110], a modification that reduces its Akt-mediated phosphorylation. In addition, FOXO1 appears to be phosphorylated by ATM/ATR [111] while FOXO3 is itself important for activation of ATM/ATR [112], [113]; these modifications may be included in future versions of the model. Updating of the model would currently be done manually, though the process would be simplified by the work already done in extracting relevant data from publications and developing fitting protocols. Moreover, parameters of processes not related to the PTM to be added or modified should remain unchanged. However, a way of computationally ascertaining the correct simplifications of the PTM set would clearly be desirable, and research is proceeding currently that addresses related problems. This has been mainly done on genetic and metabolic networks. In metabolic networks important simplifications arise because the behaviour of steady-states is studied rather than kinetics, and there are stoichiometric constraints on the reactions. Attempts have already been made to generate genome-scale models [114], [115], and even to do so automatically using annotations [116], [117]. Applying similar approaches to these to kinetic modelling may become feasible in the near future, though this will require both theoretical developments [118] and high throughput experiments with data generated in machine-readable formats [119]. One way of developing the FOXO model automatically would be, given some sets of data and a trial model, to use a Monte Carlo approach to alter that model by adding or deleting categories of PTMs and re-allocating reactions between them, and then re-fitting to optimize parameters within each model. Similar approaches have been tried to investigate the robustness of gene regulatory networks in simulations of evolution [120].

Similar approaches to generating multiple chemical species by enumerating combinations of PTMs have been implemented several times previously (Cellerator [121] StochSim [122] BioNetGen, [123]). The approach adopted here to the problem of modelling a molecule with multiple modifications, and estimating parameters in such models with many species, is similar to that of “rule-based modelling” (see papers by Hlavacek et al. [93], [124] and Faeder et al. [125] and references therein), and a similar method of reducing multiple modifications to a single collective modification has also been used previously [126]. We remark that multistate species (i.e. addition of multiple PTMs to a base species) will be supported by the non-core SBML Level 3 package “multi” which will be of great benefit for this type of modelling. Developments in rule-based modelling particularly may also be relevant to the question of updating the model, and indeed new algorithms that allow reactions to be generated as required “on-the-fly” rather than enumerated beforehand may enable the lifting of the requirement that multiple modifications be combined [127], [128].

The scripting/rule-based modelling approach to generating reactions is applicable to other proteins that undergo multiple regulations in a similar manner, for example the insulin receptor substrate IRS-1 [129]–[131] and the *C elegans* transcription factor SKN-1 (orthologous to NRF TFs in higher organisms) [132]. Following on from this, we remark that several other TFs are indeed, like FOXO, regulated by multiple PTMs, including the tumour suppressor p53 (UNIPROT accession P04637 for the human protein), which can undergo modifications including phosphorylation (by multiple kinases including ATM/ATR), acetylation, methylation and ubiquitylation at least 17 sites [133]. There have been many other papers [70], [71], [134], [135] modelling aspects of the behaviour of p53, though these have mostly concentrated on the negative feedback control through its transcription of the ubiquitin ligase MDM2 and resulting degradation, which can produce oscillations in protein concentration. p53 is usually represented in these models by more than one species, which may correspond to different PTM states. For example, in the papers by Proctor et al. and by Puszynski et al. there are both an inactive and a transcriptionally active form of p53, the latter phosphorylated in response to DNA damage. These can be viewed as omitting certain modifications that are irrelevant to the particular purpose of the model and considering the others as a single collective modification, in a similar way to how the multiple Akt-modifications of FOXO were treated here. However, to understand other aspects of the tumour suppressor function of p53, for example the interplay of responses to DNA damage and hypoxia, the interaction of multiple PTMs may be important and could be tackled by modelling using methods similar to those employed here.

To summarize, this FOXO model provides a framework into which future measurements can be fitted, and provides a central component for more complex models integrating the multiple upstream pathways and the downstream events that follow from the transcription of the multiple FOXO-regulated genes. We have demonstrated that, even alone, it demonstrates complex spatio-temporal regulations: in particular, the behaviour shown in Figure 6 may have implications for stress-resistance transcription. If FOXO is not acetylated, the long-time activation of Akt by insulin-like signalling may lead to sufficient degradation of FOXO that recovery of FOXO mediated stress-resistance transcription would be slow (in the simulation in Figure 6, this follows simply from Akt deactivation; but it could also be the result of a JNK activation to override the Akt, as in Figure 5). However, if the FOXO is simultaneously protected by acetylation, FOXO is degraded much less and transcription recovers more rapidly. In the current model, the absolute level of transcription from acetylated FOXO was slightly slower, but this could be a gene-specific effect. In combination with detailed models of the upstream and downstream pathways, this work will, we anticipate, provide insight into the interplay of ageing, nutrition and stress, and form the basis for a modelling approach connecting metabolic pathways relating to ageing, energy usage and stress with life history theory. This model represents an essential first step.

## Supporting Information

Dataset S1 Multiple sequence alignment of FOXOs from Human, Mouse, Xenopus, Zebrafish, Drosophila and C elegans (Clustal format (aln)).
(0.02 MB TXT)

Dataset S2 Python script used to generate SBML-shorthand for the models in this paper. See comments at the top of the file for usage, software requirements, and how to modify.
(0.03 MB TXT)

Dataset S3 SBML model file for the simulation in figure 4C (blue line) - Akt high, normal synthesis/degradation.
(0.06 MB XML)

Dataset S4 Sensitivity analysis (tab-delimited file) for a similar model to S3.xml (Akt = 1e5 not 2.5e4).
(0.02 MB TXT)

Dataset S5 SBML model file for the simulation in figure 4D (blue line) - Akt high, cbp high, synthesis inhibited.
(0.12 MB XML)

Dataset S6 Sensitivity analysis (tab-delimited file) for model S5.xml.
(0.07 MB TXT)

Dataset S7 SBML model file for the simulation in figure 4E - Akt low, IKK high, normal synthesis/degradation.
(0.13 MB XML)

Dataset S8 Sensitivity analysis (tab-delimited file) for model S7.xml.
(0.07 MB TXT)

Dataset S9 All above supporting information (S1–S8) in single zip archive: multiple sequence alignment of FOXOs (aln), python script, sbml models and sensitivity analyses.
(0.07 MB ZIP)
